# Laboratorial significance of autoantibodies of dense fine speckled pattern

**DOI:** 10.1186/1546-0096-10-S1-A121

**Published:** 2012-07-13

**Authors:** Tania Caroline Castro, Daniela Petry Piotto

**Affiliations:** 1 Federal University of São Paulo, Sao Paulo, Brazil

## Purpose

Indirect immunofluorescence (IIF) in Hep-2 cells is the standard screening test for antinuclear antibodies (ANA). Advances in this methodology have brought up a considerable increase in sensitivity and consequently a decrease in its specificity. This has resulted in an increasing number of positive tests in apparently healthy subjects. In particular, autoantibodies associated with the dense fine speckled (DSF) ANA-Hep-2 pattern, has been largely detected in apparently healthy individuals.

## Methods

ANA-Hep-2 test from 921 children aged 0.2 to 13.9 years was retrospectively retrieved from the routine of a large private laboratory on-site certified by the College of American Pathologists (CAP). Criteria for patient selection included concomitant existence of at least, one or more of the following tests (complete blood count, erythrocyte hemossedimentation rate [ESR], C - reactive protein [CRP], ferritin, protein electrophoresis and urinalysis). The frequency of ANA-Hep-2 patterns was analyzed according to alterations in those exams. Statistical analysis included Fisher’s exact test, Analysis of Variance (ANOVA), Kruskal-Wallis test, Mann-Whitney test with Bonferroni correction and Kolmogorov-Smirnov test.

## Results

ANA-Hep-2 was positive in 38% (350/921) of the total children, and the frequency of DFS pattern was observed in 13.4% (123/921) of the total children and in 35.1% (123/350) of those with ANA-Hep-2 positive test (Table 1). DFS pattern was significantly correlated with higher levels of CRP (p<0.01) and lymphocytes (p<0.02), although, in normal levels.

**Table 1:** Immunofluoresence patterns of antinuclear antibodies

ANA	N	%
Total	921	100.0
Non Reagent	571	62.0
Homogenous/Specified	9	1.0
Dense Fine Specified	123	13.4
Quasi-homogenous	37	4.0
Centromere	8	0.9
Others	173	18.8

## Conclusion

The DFS pattern was a frequent finding in the ANA-Hep-2 routine and its occurrence was not associated with abnormalities in general laboratory tests. This finding supports the notion that the autoantibodies associated with the DSF ANA-Hep-2 pattern are common in individuals with no apparent health abnormality.

## Disclosure

Tania Caroline Castro: None; Daniela Petry Piotto: None.

